# Trigeminal Neuralgia: A Condition to Be Better Understood

**DOI:** 10.5812/kowsar.22287523.3492

**Published:** 2012-01-01

**Authors:** Felipe Porto

**Affiliations:** 1 University of Connecticut, School of Dental Medicine, Division of Behavioral Sciences and Community Health, Farmington, USA

**Keywords:** Trigeminal Neuralgia, Patients, Multiple Sclerosis

Dear Editor, 

I read the paper entitled “Trigeminal Neuralgia: Frequency of Occurrence in Different Nerve Branches”, published in your journal (1). The data presented confirms the findings of previous papers (2-4), as was also mentioned by the author. Such findings help the clinicians to diagnose the disease. However, some other points need to be mentioned; even though this study reports no case of bilateral trigeminal neuralgia, previous publications reported that it may affect 1-5% of the patients (5, 6), and another author mentioned that bilateral cases should suggest a systemic involvement, such as multiple sclerosis (MS) or cranial tumor (7). We can conclude that bilateral cases are rare, but not impossible; and a clinician should be suspicious of a systemic condition when examining a patient with bilateral trigeminal neuralgia (TN). Considering that TN is a clinical diagnosis, the clinician should not only rely on the epidemiologic data, even though it might help on the differential diagnosis. Clinical characteristics should be considered during the history taking and clinical examination, and those characteristics can be found on the diagnostic criteria published by the International Headache Society (IHS). The IHS divides TN in two subsets:

- Classical TN, which is not related to any pathology, but vascular compression.
- Symptomatic TN, which is caused by an identifiable lesion or pathology.

It is known that over 85% of the cases with TN have the classical TN. All clinicians involved on the management of pain patients should be familiar with the diagnostic criteria published by the IHS, which helps to correctly identify patients with TN, as well as differentiate between classical TN and symptomatic TN (8).

Although diagnosing TN appears to be easy, many patients have undergone unnecessary dental treatment (9), and about 90% of patients diagnosed with TN experienced pain for more than 1 year before receiving the correct diagnosis (10).

Before establishing a long-term treatment, the health care provider should first try to identify the etiology of the TN. As the Bangash’s paper well mentioned, the clinician should closely follow the patients up, in order to select the best treatment, watch for side effects and often evaluate the patient for any change on the medical history (including new intra and extra oral exam). Clinicians should become familiar not only with treatment options, indications and contraindications for each treatment modality, side effects and adverse reactions associated with the treatment, but also with new studies about diagnosis and treatment of TN.

As all the other neuropathic pain conditions, TN is not completely understood yet. Finally, further investigations should focus not only on the treatment of TN, but also on the pathogenesis of this condition. Epidemiologic studies as the article published by Bangash on your journal are the first step to develop more specific studies regarding etiology and new treatment options in order to decrease/eliminate the patient’s suffering and clinician’s frustration.
